# Multiphasic Heterogeneity of Fibroblasts in the Microenvironment of Pancreatic Ductal Adenocarcinoma: Dissection and the Sum of the Dynamics

**DOI:** 10.3390/cancers14194880

**Published:** 2022-10-05

**Authors:** Hideaki Ijichi

**Affiliations:** 1 Clinical Nutrition Center, Graduate School of Medicine, The University of Tokyo, Tokyo 113-8655, Japan; hideij@g.ecc.u-tokyo.ac.jp; Tel.: +81-3-3815-5411; 2 Department of Gastroenterology, Graduate School of Medicine, The University of Tokyo, Tokyo 113-8655, Japan

**Keywords:** pancreatic ductal adenocarcinoma (PDAC), tumor microenvironment (TME), cancer-associated fibroblast (CAF), extracellular matrix (ECM), heterogeneity, plasticity, subTME

## Abstract

**Simple Summary:**

Pancreatic ductal adenocarcinoma (PDAC), the most common type of pancreatic cancer, characteristically exhibits abundant stroma, including marked fibrosis in the tumor microenvironment, which is considered highly associated with its crucial malignant potential and extremely poor prognosis. To conquer PDAC, it is vital to understand the microenvironment in detail. In this Special Issue, “Tumor Microenvironment and Pancreatic Cancer”, various aspects of microenvironment are discussed: heterogeneity of cancer-associated fibroblasts (CAFs), including possible cancer-promoting and -restraining properties, the significance of the extracellular matrix (ECM) and targeting ECM components, the dynamics of the immune microenvironment, metabolic property of PDAC cells in the tumor microenvironment, the association of the PDAC microenvironment and microbiome, as well as neural signaling, and the effect of anesthetic drugs on PDAC. This evidence indicates that the PDAC microenvironment is more complicated than expected. The suggested multiphasic diversity and heterogeneity of the PDAC microenvironment may be a consequence of the sum of more transient change and dynamic plasticity through various tumor–stromal interactions.

**Abstract:**

Pancreatic cancer is still the most intractable cancer, with a 5-year survival of around 10%. To conquer the most common type, pancreatic ductal adenocarcinoma (PDAC), we need to understand its pathobiology, especially the tumor microenvironment (TME) that characteristically contains abundant stromal components, with marked fibrosis. In this Special Issue, “Tumor Microenvironment and Pancreatic Cancer”, various aspects of TME were discussed, most frequently including articles related to cancer-associated fibroblasts (CAFs) and the extracellular matrix (ECM). CAFs and ECM have been considered in favor of PDAC cells; however, surprisingly, depleting CAFs or reducing the stromal components in PDAC-model mice induced aggressive PDAC and worsened the prognosis. Subsequently, accumulating studies have elucidated evidence of the heterogeneity of CAFs and the plasticity between the subtypes. Possible cancer-promoting and -restraining properties of the CAF subtypes have been suggested, but these are yet to be fully elucidated. Here, in addition to the extensive reviews on the heterogeneity of CAFs in this Special Issue, I refer to another insight from a recent integrative study of PDAC TME, that PDAC TME can be divided into three distinct sub-tumor microenvironments (subTMEs), and the co-existence of the distinct subTMEs is associated with poor prognosis. In the subTME, the heterogeneity of each component, including CAFs, can be changed transiently through various interactions in the TME, and the sum of the transient change and dynamic plasticity might be timely tuned in the co-existence of distinct subTMEs to contribute to the poor prognosis. Thus, understanding the more detailed underlying mechanisms in this heterogeneity of TME, as well as how to control the sum of multiphasic heterogeneity, might lead to the establishment of a more desirable therapeutic strategy to conquer intractable PDAC.

## 1. Introduction

In the last two decades, cancer therapies have advanced significantly: novel therapeutics, including molecular targeting therapies and immune checkpoint inhibitors, have dramatically changed clinical practice and improved the prognosis in many cancer types. Enormous efforts have been made regarding pancreatic cancer as well; however, its 5-year survival rate is still around 10%, which has been slightly elevated over the last few decades, but remains devastatingly poor compared to other cancers [1,2]. The incidence and mortality of pancreatic cancer are increasing year by year, and it is expected to be the second-leading cause of cancer death in the United States by the year 2030 [1,2,3]. Therefore, improving the prognosis of pancreatic cancer, especially of the most common type, pancreatic ductal adenocarcinoma (PDAC) is one of the ultimately unmet medical needs.

One of the important reasons for the poor prognosis of PDAC is its histological characteristics. In the PDAC tissues, cancer cells are few, and most of the tissues are occupied by abundant stromal components, such as fibroblasts, immune-inflammatory cells, blood and lymphatic vessels, neural cells, the extracellular matrix, etc., all of which form the PDAC tumor microenvironment (TME), presenting marked fibrosis, called desmoplasia [4,5]. The “desmoplastic” cancers, such as PDAC, scirrhous gastric cancer, and inflammatory breast cancer, are uniformly known as poor-prognostic types; therefore, it is believed that the marked fibrosis in the TME is associated with a poor prognosis [5,6,7]. In addition to the location of the pancreas, which cannot be easily and routinely approached by diagnostic screening, the histological features of the paucity of cancer cells, related to the indistinct boundary of tumor nodules, might also explain the difficulty of early diagnosis of PDAC: 80% of the patients cannot undergo curative surgical resection at diagnosis. However, even if radical resection is successfully performed, PDAC has a high frequency of recurrence and shows chemoresistance, which suggests the biologically malignant potential of PDAC, which might also be highly associated with the characteristic TME [4,5]

The dense stroma and fibrosis of PDAC TME has been considered as a physiological barrier to protect PDAC cells from the chemotherapeutic drugs and anti-tumor immunity by collapsing vessel cavities and inhibiting immune cell infiltration. Genetically engineered mouse models (GEMMs) of PDAC have been established in the context of pancreas epithelium-specific mutant Kras knock-in, which can histologically recapitulate human PDAC, containing “intact” TME [8,9,10,11,12], and the barrier function was also confirmed: reduced drug delivery into the tumor nodules in GEMM was shown, compared to the transplanted tumors, and enhanced drug delivery extended the survival of the mice [13].

However, two striking studies reported that the depletion or reduction in the barrier components in GEMMs induced the aggressiveness of PDAC and worsened the GEMM survival [14,15], which suggested that there might be a cancer-inhibiting function in the PDAC TME; this was a pivotal concept change. Subsequently, the diversity and heterogeneity in the PDAC TME have attracted attention and been intensively studied. Recently, next-generation sequencing, single-cell analyses, and multi-omics analyses have progressively collected information by using GEMMs, as well as patient tissues and patient-derived models. Distinct clusters of a variety of non-cancer cells, possibly with pro-tumor and anti-tumor functions, have been discovered; therefore, a more comprehensive understanding of the complicated TME is required for the establishment of a therapeutic strategy for regulating and conquering PDAC.

In this Special Issue, “Tumor Microenvironment and Pancreatic Cancer”, various aspects of PDAC TME are discussed: the heterogeneity of cancer-associated fibroblasts (CAFs), including cancer-promoting and cancer-restraining properties [16,17,18], the significance of the extracellular matrix (ECM) and targeting the ECM components [19,20,21], the dynamics of the immune microenvironment, [22,23], the metabolic property of PDAC cells in the TME [24], the association of PDAC TME and the microbiome [25], as well as neural signaling, [26], and the effect of anesthetic drugs on PDAC [27]. This evidence indicates that the PDAC microenvironment is more complicated than expected.

The transcriptional and functional heterogeneity of CAFs is the most frequently discussed theme in this Special Issue [12,13,14]. It is highly complicated and still somewhat controversial. In addition to the reviews, here, I refer to another insight from a recent integrative study of PDAC TME, that PDAC TME can be divided into three distinct sub-tumor microenvironments (subTMEs), and the co-existence of the distinct subTMEs is associated with a poor prognosis. In the subTME, the heterogeneity of each component, including CAFs, can be changed transiently through various interactions in the TME, and the sum of the transient change and dynamic plasticity might be timely tuned in the co-existence of distinct subTMEs to contribute to poor prognosis. Thus, understanding the more detailed underlying mechanisms in this heterogeneity of TME, as well as how to control the sum of multiphasic heterogeneity, may lead to the establishment of a more desirable therapeutic strategy to conquer intractable PDAC.

## 2. The PDAC Microenvironment Consisted of Diverse Cell Populations and Extracellular Matrix Proteins in Favor of PDAC Cells

As shown in the comprehensive review by Skorupan et al. [20], TME consists of a number of different types of cells and non-cellular, extracellular matrix ECM proteins, creating a diverse microscopic ecosystem. PDAC TME is characteristic by abundant stroma and a low percentage of cancer cells. CAFs play central roles in the stroma, producing ECM proteins and forming fibrosis, thereby inducing high interstitial pressure, with collapsed vessel cavities and a hypovascular condition. Inflammation has been proved to accelerate PDAC formation, and the immune-inflammatory cell infiltration is observed from an early stage of carcinogenesis [28,29]. Thus, there are a number of different types of cells—tumor cells, CAFs, neutrophils, macrophages, myeloid-derived suppressor cells (MDSCs), various T and B lymphocytes, neural cells, vessel endothelial cells, pericytes, etc.—communicating with each other to establish the PDAC TME. Through the numerous tumor–stromal interactions, the stromal cells are cultivated by cancer cells, resulting in the TME in favor of cancer cells, creating a difficult condition for the chemotherapeutic drugs to reach the cancer cells and also an immunosuppressive condition for PDAC cells, allowing them to escape from the anti-tumor immune surveillance [20]. The heterogeneity of the neutrophils and macrophages has previously been recognized; through the tumor–stromal interactions, the proportion of neutrophils and macrophages changes to enrich tumor-associated neutrophils (TANs) and tumor-associated macrophages (TAMs), forming the immunosuppressive condition favorable for cancer cells [22,30]. Among the T cell population, regulatory T cells (Treg) also contribute to the immunosuppressive TME [22]. 

Thus, the chemoresistant and immunosuppressive stromal barrier has been considered as a potent therapeutic target, and blocking the tumor–stromal interaction or reducing the stromal components have been attempted. In line with this, we have investigated the tumor–stromal interactions between PDAC cells and CAFs in GEMM and found that PDAC cells and CAFs produced and secreted the same kinds of CXC chemokines (ligands of the CXC chemokine receptor 2 (CXCR2)), and CAFs enhanced the PDAC cell invasion, while PDAC cells enhanced the CAF migration, suggesting that PDAC cells and CAFs might attract each other and contribute to their invasion and metastasis [31,32]. Of note, the CXC chemokines did not directly affect the cell proliferation of PDAC cells and CAFs; however, mixed transplantation of PDAC cells and CAFs enhanced in vivo tumor growth compared to that of PDAC cells alone, indicating the cancer-promoting tumor–stromal interaction. Treating GEMM with a CXCR2 inhibitor, or the systemic knockout of CXCR2 in GEMM in our study and others, significantly extended the survival of the mice, indicating an impact on the prognosis [31,32,33]. These examples are consistent with the idea that CAFs are cancer promoting in PDAC and indicate that they function as more than just barriers.

## 3. CAFs and Fibrosis in the PDAC Microenvironment Are Not Simply Cancer Promoting

In 2014, two epoch-making studies argued against the idea that CAFs and fibrosis are simply cancer promoting. Özdemir et al. depleted α-smooth muscle actin (α-SMA)-positive CAFs in GEMM, because α-SMA-positive CAFs had been considered as activated CAFs through the tumor–stromal interactions, with possible expectation that the depletion of α-SMA-positive CAFs would reduce the fibrosis, as well as stromal volume, and deplete the cancer-promoting activities, resulting in improved prognosis. However, the tumor became a poorly differentiated type and the survival of the mice was significantly reduced. They also showed poor prognosis in the high α-SMA expression group of human PDAC patients after surgery [14]. Rhim et al. inhibited hedgehog signaling in GEMM, which is highly activated in CAFs and involved in enhanced fibrosis, but also observed the poorly differentiated tumors and shortened survival time of the mice, although the stromal volume was reduced [15].

These two studies have changed the previously common belief that CAFs in PDAC were uniformly cancer-promoting and the dense stromal barrier should be simply depleted. However, PDAC TME was more complicated than expected, and CAFs were not just cancer-promoting. Subsequently, the concept of heterogeneity of CAFs has emerged and has been intensively investigated, as described in detail by Masugi [16], Ando et al. [17], Shinkawa et al. [18], and others in this Special Issue. The two studies indicated that the stroma has a role in inhibiting PDAC progression, with the regulation of histological differentiation. CAFs and fibrosis are not only cancer-promoting; thus, they cannot be simply depleted or inhibited, and fibrosis can function as a barrier for the host side, preventing PDAC from progression. Both studies showed a reduction in stromal volume and suggested a possibility to improve the prognosis in certain combinations with chemotherapy or anti-angiogenic therapy by increasing drug delivery [14,15]; however, clinical trials targeting stromal hedgehog signaling or hyaluronan acid, in combination with chemotherapies, revealed no significant benefit or harm [34,35], which also supported the idea that CAFs and fibrosis have tumor-inhibiting roles.

The concept has changed, and we have realized that we need to understand the heterogeneity of CAFs; however, the characterization of cancer-promoting CAF and cancer-restraining CAF still remains to be fully elucidated. To date, no novel therapy directly targeting the CAF and fibrotic TME has been established, but clinical trials targeting stroma and ECM molecules in PDAC have been carried out and are ongoing, as Wang et al., Skorupan et al., and Masugi described [16,19,20]. If the effects of these molecular targeting approaches on the heterogeneity of CAFs are more precisely elucidated, it might provide an additional proof for the therapies and clearer focus for the trials. 

## 4. Transcriptional and Functional Heterogeneity of CAFs in PDAC Microenvironment

Along with significant progress in the next-generation sequencing (NGS) technology, comprehensive genome and transcriptome sequencing of the PDAC epithelium was performed, and the transcriptional classification of PDAC was reported by several groups [36,37,38]. According to those reports, two major molecular subtypes of PDAC epithelial cells are now widely accepted: basal-like/squamous subtype, and classical subtype. Basal-like/squamous PDAC is reported to be more aggressive and have a poorer prognosis compared to classical PDAC. It was also reported that the epithelial transcriptional PDAC phenotype is formed through the tumor–stromal interaction, not solely by the epithelia [37].

The heterogeneity of CAFs was also identified, and knowledge has been acquired through further advances in NGS using single-cell analysis, multiple immunostainings, and multi-OMICs analyses.

### 4.1. myCAF and iCAF: Two Current Dominant CAF Clusters

**Figure 1:**
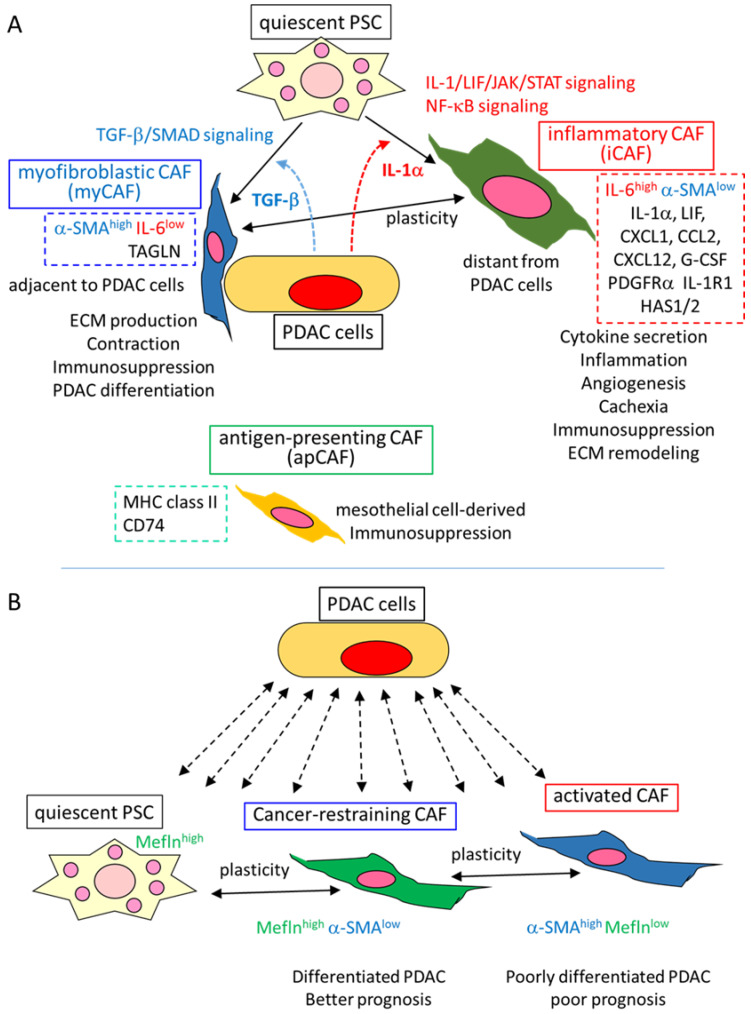
Functional heterogeneity and plasticity of CAFs in PDAC. (**A**) Three CAF clusters identified by Tuveson’s group and their suggested functions [39,40,41]. Through the interaction with PDAC cells (and others) in the TME, quiescent PSC is converted to two CAF subtypes, depending on distinct signals induced by PDAC cells. Myofibroblastic CAF (myCAF) is induced by TGF-β/SMAD signaling at the location adjacent to PDAC cells. myCAFs express high α-SMA and TAGLN, but low IL-6, and secrete ECM proteins, indicating a myofibroblast-like subtype. Inflammatory CAF (iCAF) is induced by IL-1α/LIF/JAK/STAT signaling, as well as NF-κB signaling at a location distant from PDAC cells. iCAFs express high IL-6 and low α-SMA, and also express many kinds of cytokines, as well as PDGFRα, IL-1α, and HAS1/2, suggesting that iCAFs are involved in inflammation and other TME conditions by secreting those cytokines, as well as in ECM remodeling by HAS1/2. The plasticity is shown between myCAF and iCAF through the balance of the signaling of TGF-β and IL-1α. Antigen-presenting CAF (apCAF), expressing MHC-II and CD74, is reported to be mesothelial cell-derived. All these subtypes may contribute to an immunosuppressive TME condition. (**B**) Meflin expressing cancer-restraining CAF and plasticity, along with the expression of a-SMA and Meflin, as determined by Mizutani et al. [42]. Through the interaction with PDAC cells (and others) in the TME, quiescent PSC is converted to Meflin^high^ α-SMA^low^ CAF, and subsequently, to α-SMA^high^ Meflin^low^ CAF. CAF subtypes might affect PDAC differentiation and prognosis. Meflin^high^ α-SMA^low^ CAF is reported to be cancer-restraining.

Tuveson et al. firstly identified three distinct clusters of CAF in PDAC GEMM, as well as human PDAC: myofibroblastic CAF (myCAF), inflammatory CAF (iCAF), and antigen-presenting CAF (apCAF) [39,40,41] (Figure 1A).

As Shinkawa et al. and Masugi described this clustering in detail [16,18]; myCAF was identified as a cluster expressing high α-SMA and low interleukin (IL)-6, located adjacent to PDAC cells, and containing myofibroblastic characteristics of contraction, producing ECM proteins. The property was TGF-β-SMAD signal-dependent, and CTGF (connective tissue growth factor), TAGLN (transgelin) and other fibroses, ECM, and contraction-related molecules were all upregulated. On the other hand, iCAF was identified as a cluster, with high IL-6 and low α-SMA expression, and was found to be distantly located from the PDAC cells. iCAF expressed a number of inflammatory cytokines and chemokines, including IL-6, IL-1α, CXCL1, and CCL2, as well as the other secreted factors granulocyte-colony stimulating factor (G-CSF), CXCL12, and leukemia inhibitory factor (LIF). Platelet-derived growth factor receptor α (PDGFRα), IL-1 receptor 1 (IL-1R1), and HAS1/2 (hyaluronan synthase 1/2) were also differentially expressed in iCAF compared to myCAF. These differentially expressed genes were considered to be the specific markers of each CAF cluster. The secretory phenotype of iCAF was reported to be induced and maintained by IL-1/LIF/JAK/STAT and NF-κB signaling [39,40]. 

The iCAF and myCAF were shown to be induced from pancreatic stellate cells (PSCs) in distinct culture conditions with PDAC cells: myCAF was induced by contact co-culture of quiescent PSC with the PDAC organoid, while iCAF was induced in the separated co-culture using transwell cell culture assays, which was consistent with the location in the tumor tissue, as described above, and considered as a paracrine of TGF-β and IL-1α from the PDAC cells. Indeed, IL-1α treatment increased iCAF marker molecule expression and decreased myCAF marker expression in PSC, whereas TGF-β treatment decreased IL-1R1 expression and iCAF marker expression in PSC, indicating a plasticity between PSC and the CAF types, as well as a mutually exclusive property between iCAF and myCAF, with the underlying mechanism of antagonism between IL-1/JAK/STAT and TGF-β signaling [39,40]. This was also supported in vivo; GEMM treated with TGF-β inhibitor showed a significant decrease in collagen deposition and α-SMA expression, with an increase in iCAF content, which suggested the inhibition of myCAF, while treatment with JAK inhibitor significantly increased collagen deposition and α-SMA expression, with a shift from iCAF to myCAF population, which suggested the inhibition of iCAF. Of note, the JAK inhibitor significantly decreased the PDAC tumor volume, whereas the TGF-β inhibitor did not change the tumor volume [39,40]. 

Since the depletion of α-SMA-positive CAF worsened the PDAC phenotype and prognosis [14], myCAF was considered as a cancer-restraining CAF, whereas the distinct cluster iCAF was considered as a cancer-promoting CAF because the inflammatory cytokines and other factors secreted by iCAF have been shown to promote PDAC, and also due to the PDAC-suppressing effect of JAK/STAT inhibition, described above [40]. Similar iCAF and myCAF clusters were repeatedly identified in PDAC, as well as in other cancer types, by several single-cell RNA sequencing analyses [43,44], as described by Shinkawa et al. [18]. Therefore, these two CAF clusters have been considered as the dominant clusters of CAF.

### 4.2. apCAF and Other CAF Clusters

The third cluster of CAF, apCAF, was also identified by a single-cell RNA sequence in GEMM, which expressed molecules involved in antigen presenting, such as major histocompatibility complex (MHC) class II and CD74 [41]. However, apCAF did not express co-stimulatory molecules; thus, its tumor immunity and other functions still remain unclear.

After the first documentation of the three CAF clusters, other CAF clusters were also reported, with or without iCAF, myCAF, and apCAF [44,45]. Initially, iCAF and myCAF were induced from quiescent PSC; however, a recent study documented that PSC-derived CAFs are only a minor fraction of the total CAFs in PDAC [46]. As Masugi also described [16], previous reports have indicated that CAF can be derived from multiple cell origins, including resident fibroblasts, bone-marrow-derived mesenchymal stem cells, adipose-derived stem cells, mesothelial cells, endothelial cells, and epithelial cells [47]. The apCAF was recently suggested to be of mesothelial cell origin [48]. Thus, the heterogeneous cell origin of CAFs might contribute to the heterogeneity of CAF clustering, suggesting that accumulating studies using other models and cohorts might further elucidate different novel CAF clusters or novel subclusters derived from existing CAF clusters.

### 4.3. Heterogeneic Functions of CAFs in PDAC

Since the identification of the two dominant CAF clusters, differential functions of the CAF subtype have been expected in the context of cancer-promoting iCAF and cancer-restraining myCAF. Although the secretory phenotype of iCAF and the ECM-producing property of myCAFs were well characterized by molecular expression profiles [39,40,41], the evidence of cancer-promoting and cancer-restraining properties of each CAF subtype does not seem to be fully elucidated. 

The cancer-promoting functions of CAFs have been identified in various cancers, including PDAC. Moreover, as described in several reviews in this Special Issue [16,17,18,20], CAF can promote cancer progression in multiple ways, e.g., (1) secreting ECM proteins; (2) inducing inflammation; (3) increasing angiogenesis; (4) establishing an immunosuppressive TME; (5) affecting the signaling of cancer cells; (6) changing the metabolism of cancer cells; (7) establishing chemo-and radio-resistant TME; (8) promoting tumor invasion and metastasis; (9) secreting pro-tumorigenic ligands, etc. [49], and many of these are overlapping; for example, inflammation induces immune-inflammatory cell infiltration and forms immunosuppressive TME. 

Most of these functions are associated with cytokines, chemokines, and other factors secreted by CAFs, e.g., IL-6, IL-1α, CXCL1, CCL2, CXCL12, and G-CSF, which suggests the contribution of iCAF. However, which CAF subtype is really secreting these factors, and whether the CAF is the most responsible cell type among the various TME cell components, still remains to be fully determined. ECM deposition and dense stroma are characteristics of PDAC TME, which also promote PDAC in multiple ways. The dense ECM stroma acts as a physical barrier to drug delivery and also forms immunosuppressive TME, and the high tissue tension and stiffness induces PDAC cell invasion and metastasis [50]. Producing ECM proteins is considered as a characteristic of myCAF, but iCAF also produces collagen 1A1 and other ECM proteins, as well as ECM-remodeling enzyme HAS1/2 [41]. Which CAF subtype is responsible for the cancer-promoting effect of ECM also remains to be elucidated. Immunosuppressive TME consists of various immune cell types, TAMs, TANs, MDSCs, Tregs, etc. CXCL12 was reported to induce immunosuppressive TME in PDAC, which might be secreted by iCAFs. In addition, TGF-β is a well-known driver of immunosuppressive TME in many cancers, as it inhibits anti-tumor T cell and NK cell function, as well as induces Treg differentiation [51]. Considering that myCAF is induced and maintained by TGF-β signaling, the immunosuppressive TME might be established by the cooperation of iCAF and myCAF. apCAF was also reported to induce Treg [48]; thus, all three CAF subtypes might contribute to the immunosuppressive TME.

### 4.4. Recent Findings of Functional Heterogenity of CAF Subtypes

Accordingly, the CAF subtype-specific cancer-promoting or cancer-restraining function remains to be fully elucidated. Recently, several studies documented or suggested the functional difference of CAF subtypes regarding cancer progression. Dominguez et al. identified CAF clusters in PDAC and other cancers, including iCAF, myCAF, and apCAF, and revealed that TGF-β-induced myCAF signature was correlated with poor prognosis in urothelial cancer patients with anti-PD-1 (programmed cell death 1) therapy [44], which suggests that myCAF might be associated with resistance to immune therapy. McAndrews et al. reported that depleting FAP (fibroblast activation protein)-expressing CAFs prolonged the survival of PDAC GEMM, whereas the depletion of α-SMA-expressing CAFs shortened their survival. Moreover, selective IL-6 ablation in α-SMA-expressing CAFs improved gemcitabine efficacy, as well as their synergy with anti-PD-1 therapy [52]. This study suggests that CAFs are cancer promoting as a whole, and myCAFs are cancer restraining, but there might be a cancer-promoting subcluster among the myCAFs. Steele et al. observed that the inhibition of hedgehog signaling decreased PDAC tumor volume, but in the TME, an increase in iCAF and decrease in myCAF was observed, accompanied by a decrease in CD8^+^ T cells and an increase in Tregs, suggesting a more immunosuppressive TME [53]. Pan et al. co-cultured PDAC cells with mesenchymal stem cells (MSCs) and found that PDAC cells with liver metastasis potential specifically induced the iCAF signature in MSCs, but PDAC cells with lung metastasis potential did not, suggesting CAF subtype-specific metastasis niche formation [54]. Li et al. identified four CAF clusters in human gastric cancer and found that distinct CAF subtypes interact with different immune cell populations to form immunosuppressive TME: iCAF induces lymphocyte recruitment and regulates CD8^+^ and PD-1^+^ T cells by secreting IL-6 and CXCL12, while periostin expressing another CAF subtype recruits M2 macrophages. Periostin expression was significantly associated with poor prognosis in gastric cancer patients [55]. Hu et al. integrated a single-cell RNA sequence and survival analysis from the public PDAC database, with validation by multiple immunohistochemistry analyses, and observed that the myCAF signature was associated with poor prognosis, while the iCAF and apCAF signatures were associated with a better prognosis [56].

Taken together, the functional heterogeneity of CAF subtypes has been gradually elucidated; however, whether cancer promotion or cancer restraint appears is not always consistent with the initial expectation. This might be because the CAF subtypes interact with PDAC cells, as well as other cell types, through the complicated network in the TME.

### 4.5. Meflin-Positive Cancer-Restraining CAF

Mizutani et al. reported Meflin-expressing cancer-restraining CAFs [42], which is the focus of the review by Ando et al. in this Special Issue [17] (Figure 1B). Meflin is a protein that has an inhibitory effect on collagen cross-linking. They described that Meflin is highly expressed in quiescent PSC, and when the PSC is activated and converted to CAF through the interaction with the cancer cells and TME, the expression of Meflin decreases along with the increasing expression of α-SMA. Thus, Meflin-positive CAF is a subtype with low α-SMA expression. Meflin-knockout GEMM revealed poorly differentiated PDAC, increased α-SMA expression in CAFs, and shortened survival. In contrast, Meflin overexpression in CAFs decreased the α-SMA expression and inhibited the transplanted tumor growth. In the resected PDAC tissue analysis, the patients with high-Meflin CAF showed better prognosis compared to those with low-Meflin CAF. These results suggested that Meflin-positive CAF regulates differentiation of PDAC and has a cancer-restraining property [42] (Figure 1B).

Thus, CAF heterogeneity and the plasticity of quiescent PSC through the activated CAF was demonstrated yet again: the quiescent PSC, containing a lipid droplet of vitamin A, loses the lipid droplet along with decreased Meflin expression in the PDAC TME, and becomes activated CAF with high α-SMA and low Meflin expression. Related to this plasticity, it was also demonstrated that adding the vitamin D receptor ligand induced Meflin expression and decreased α-SMA in the CAFs [42]. Previously, Sherman et al. reported that vitamin D restrained quiescent PSC from PDAC-induced conversion into activated CAF, thereby inhibiting inflammation and fibrosis and increasing gemcitabine delivery into the PDAC cells, resulting in the tumor volume reduction and survival extension of GEMM [57]. Based on these results, the vitamin D analog, along with retinoid, a vitamin A-derivative, are now advanced for use in clinical trials, in combination with chemotherapy in PDAC patients, which can be used as a “CAF normalizing” or “CAF restraining” strategy, as described by Ando et al. [17].

### 4.6. Functional Significance of α-SMA-Expressing CAF: Friend or Foe?

Heterogeneous CAF subtypes, as well as the plasticity, have been identified, and evidence suggested that CAFs can be either cancer promoting or cancer restraining, depending on the signals they receive, as well as the location and the distance from the cancer cells. However, there is controversy, especially regarding the functional property of α-SMA expressing CAF—high-α-SMA myCAF, defined by Tuveson et al., might be cancer-restraining [39,40,41], while high-α-SMA and low-Meflin CAF, as determined by Mizutani et al., might be cancer promoting [42]. 

Historically, α-SMA has been considered a hallmark of activated CAF, and CAF, as a whole, has been considered to be cancer promoting [58], which is in line with the idea that α-SMA expressing CAF is cancer promoting. However, the depletion of α-SMA-expressing CAF exacerbated PDAC [14], which was explained by the depletion of the cancer-restraining CAF subtype, α-SMA expressing myCAF. The association of the α-SMA expression level and PDAC prognosis has been contrastingly reported, and α-SMA^high^ tumor has been shown to influence both better [14] and worse prognoses [59,60]. In GEMM, the deletion of type I collagen in α-SMA-expressing CAFs promoted PDAC aggressiveness and poor prognosis [61], which might support the cancer-restraining property of α-SMA expressing CAF. On the other hand, human PDAC studies reported that a high ratio of α-SMA-positive area to collagen-positive area, or α-SMA-rich stroma type, was associated with poor prognosis in the patients [62,63,64], which might support the cancer-promoting property of α-SMA expressing CAF. Recent analyses using single-cell sequencing revealed a worse prognosis of PDAC correlated with α-SMA expressing the myCAF signature, as described above [56], which might also support the cancer-promoting property of myCAF.

According to these results, α-SMA expressing CAFs, even myCAF, can be cancer promoting and also cancer restraining, as also described in Section 4.4. α-SMA expressing CAFs might function similarly by producing ECM proteins with myofibroblast-like activities, and the prognostic effect might be determined through the multiple interactions with the other components in PDAC TME.

myCAF was initially described as being located adjacent to cancer cells and reported to be induced by TGF-β derived from cancer cells to produce ECM, which might have a barrier function for the host side to restrain the cancer cells from invasion and metastasis. However, why do the cancer cells themselves induce cancer-restraining CAF at this very close location? Since TGF-β is also known to be highly produced in the stroma, there might be another explanation, e.g., that host stroma-derived TGF-β might induce myCAF to restrain cancer cells. Alternatively, cancer cells might secrete TGF-β and induce myCAF to function as cancer-promoting CAF, to obtain a niche for cancer cells and immunosuppressive TME.

### 4.7. Multi-Layered Heterogeneity of PDAC Microenvironment 

The review of Masugi provided insights into the multi-layered heterogeneity of PDAC TME [16]. (1) Heterogeneity at the niche level: heterogeneity of CAFs and the associated intra- and intercellular signaling, e.g., TGF-β and IL-1α signaling, etc.; (2) heterogeneity at the locoregional level: the heterogeneous CAF subtype and collagen distribution, which is associated with PDAC differentiation, e.g., the collagen-rich stroma correlates with the differentiated tumor, while the CAF-rich stroma correlates with the poorly differentiated tumor [64]; (3) heterogeneity at the organ level: heterogeneity of the TME between the primary tumor and the metastatic tumor in the liver or lung, even in the same patient. The review focused on CAFs and ECM, but at niche and locoregional levels; this idea is also true for every component in the PDAC TME. As described above, various cell types other than CAFs and ECM are included, and each cell type contains heterogeneity of the cell type itself, e.g., basal-like/squamous-type and classical-type PDAC cells, and M1-inflammatory and M2-immunosuppressive macrophages, and the heterogeneity is dependent on various intra- and intercellular signaling networks with various cell types in the PDAC TME. The location in the tumor is also significantly involved in the formation of heterogeneity in each cell type, which might also be included in the concept of niche.

The review of Masugi [16] also mentioned the iCAF and myCAF in human PDAC tissues by including his own data [64]. In human PDAC tissues, his group observed that CAFs tightly surrounding well-differentiated cancer ducts frequently show high α-SMA expression within the collagen-rich stroma, suggesting a myCAF-like subtype, and CAFs expressing IL-6 or FAP-α are located more distantly from the cancer ducts, suggesting an iCAF-like subtype [64], which was consistent with the concept of Tuveson et al. According to the immunofluorescence, the expression ratio of α-SMA to FAP-α appears somewhat variable [16], as is the ratio of Meflin to α-SMA, shown by Ando et al. [17], suggesting the plasticity between the CAF subtypes. The PDAC patients were divided into three distinct stroma types—collagen-rich stroma, FAP-α-rich stroma, and α-SMA-rich stroma—and the patients with collagen-rich stroma showed better prognoses compared to those with CAF-rich stroma [16]; furthermore, the patients with α-SMA-rich stroma had a poor prognosis compared to those with FAP-α-rich stroma [64]. Thus, their observation might be partially consistent with the concept of iCAF and myCAF in terms of the location and marker expression in human PDAC TME, as well as partially inconsistent, especially with the prognosis related to the stroma type.

## 5. Transient and Dynamic Heterogeneity of CAFs and Stroma in Human PDAC Sub-Microenvironment

A recent large-scale integrated analysis of histologically guided multiOMICs and clinical data of human PDAC might help us to understand the PDAC TME with another comprehensive point of view.

**Figure 2:**
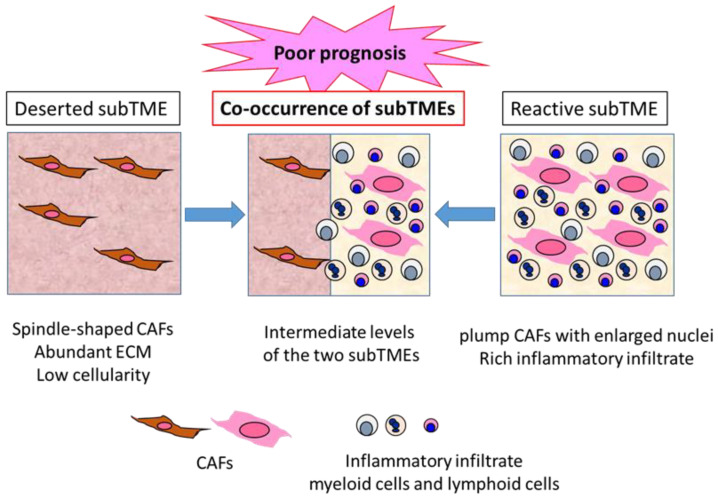
Three types of PDAC subTME and prognoses. PDAC TME can be divided into characteristic subTME, which is the sum of various components in the TME, with distinct gene and protein expression profiles. Deserted subTME contains spindle-type CAFs and abundant ECM, with low cellularity. Reactive subTME contains plump CAFs, with enlarged nuclei and rich inflammatory infiltrate. Co-occurrence of the two distinct subTMEs is frequently observed in the same PDAC tumor, indicating a poor prognosis compared to the tumors containing one major subTME.

Grünwald et al. identified three recurrent sub-tumor microenvironment (subTME) phenotypes in human PDAC: deserted regions, with thin, spindle-shaped CAFs with abundant ECM and low cellularity; reactive regions, with plump CAFs with enlarged nuclei and rich in inflammatory infiltrate; and regions with intermediate levels of these [65] (Figure 2). These subTME phenotypes frequently co-existed within the same tumors. Deserted subTME exhibited a strong enrichment in ECM, ECM pathway, and humoral immunity gene sets, while reactive subTME was enriched in gene sets related to the cellular stress response (heat shock, hypoxia, growth, metabolic stress), growth factors with CAF-activation, as well as immunomodulatory functions (FGF, TGF, PDGF, Wnt) and cellular immunity (innate immunity, antigen presentation, T cell receptor signaling). Notably, single cell analysis of isolated CAFs from deserted or reactive subTME revealed 11 distinct clusters, and the difference between the deserted and reactive subTME CAFs did not seem to match that between iCAF and myCAF [65]. There seemed to be CAF plasticity between deserted, intermediate, and reactive subTMEs. The subTME also appeared to affect the tumor cell phenotypes: the deserted subTME appeared to support tumor differentiation, while the reactive subTME promoted tumor progression through proliferative, dedifferentiated, basal-like/squamous type tumor phenotypes. Immune phenotypes of subTMEs were shown—deserted subTME was only immune-cold-like, whereas reactive subTME contained increased CD8^+^ T cells, as well as increased levels of PD-L1 (PD-1 ligand 1), FOXP3 (forkhead boxprotein 3) (regulatory T cells), CD11b (myeloid-derived suppressor cells), and CD206 (M2 macrophages), suggesting the co-existence of immune-hot, as well as immune-suppressive, components in the reactive subTME. In addition, α-SMA expression was higher in reactive subTME as opposed to deserted subTME [65]. 

These results suggest that reactive subTME might be mainly cancer promoting, while deserted subTME might be cancer restraining. However, Kaplan–Meier analysis in the patients with resected PDAC revealed that co-occurrence of deserted and reactive subTMEs revealed significantly poor prognosis compared to the patients with one major subTME. Between the major subTME groups, deserted subTME showed better disease-free survival compared to reactive subTME; however, upon chemotherapy, deserted subTME became more frequent, suggesting its chemoprotective property [65] (Figure 2). Thus, the functional interpretation of subTME might change upon chemotherapy and immunotherapy. In this Special Issue, Hegar et al. demonstrated the distinct prognostic significance of α-SMA-positive CAF density and stroma volume between the patient groups, with or without neoadjuvant chemotherapy [21], which might be consistent with this idea.

Considering these results together, it is suggested that the heterogeneity of CAFs and the co-existence of diverse subTMEs induce a much higher variety of changes in the PDAC TME, including the changes in the tumor cells and CAFs themselves, compared to the one major subTME (multi-directional changes rather than unidirectional changes), thereby timely tuning all the changes, including chemoprotective change upon chemotherapy, and thus contributing to poor prognosis. Since the plasticity of heterogeneity in CAFs, as well as subTMEs, was demonstrated, the cancer-promoting or cancer-restraining properties are not permanent, but are more transient and can be dynamically changed depending on various conditions. The sum of the transient and dynamic heterogeneity, such as the “balance of good and bad stroma”, described in Ando et al. [17], might lead to the prognostic outcomes identified. The sum includes every component in the TME and every interaction network in the TME, including immune TME, as suggested by Rubin et al. [22] and others, as an essential integrant. This might explain the inconsistency observed between the α-SMA expression and prognosis, as well as between the CAF subtype and the cancer-promoting or -restraining property.

Recent advances in the literature have successfully dissected the disease into a single-cell base, thereby determining the heterogeneity and confirming the functional cell–cell interactions. However, the interactions can be changed by their simultaneous interactions with other components in the TME, resulting in the plasticity of heterogeneity; thus, it is considered that the dissected function cannot directly account for the long-term outcomes. Therefore, we need to take two approaches, namely, dissecting the disease and characterizing its heterogeneity, evaluating the sum of both dissected findings. To investigate the sum effect, GEMM maintaining intact TME might be useful.

## 6. Conclusions

In this Special Issue, “Tumor Microenvironment and Pancreatic Cancer”, various aspects of PDAC TME were discussed, which definitely increases our understanding of the intractable cancer PDAC. Accumulated studies, along with the articles reviewed here, have elucidated diversity and heterogeneity in the PDAC TME, including its underlying mechanisms and functions. PDAC TME contains multiphasic heterogeneity, which appears more heterogeneous and more dynamic than expected, and the sum of the heterogeneous activities results in PDAC as a whole. There might still be significant challenges in conquering PDAC, but we should keep trying to understand PDAC more precisely through focusing on the TME, which is a crucial determinant of the fate of PDAC.
